# Recent CO_2_ rise has modified the sensitivity of tropical tree growth to rainfall and temperature

**DOI:** 10.1111/gcb.15092

**Published:** 2020-05-22

**Authors:** Pieter A. Zuidema, Ingo Heinrich, Mizanur Rahman, Mart Vlam, Sophie A. Zwartsenberg, Peter van der Sleen

**Affiliations:** ^1^ Forest Ecology & Forest Management Group Wageningen University Wageningen The Netherlands; ^2^ Section Climate Dynamics and Landscape Evolution GFZ German Research Centre for Geosciences Telegrafenberg Germany; ^3^ Geography Department Humboldt University Berlin Germany; ^4^ Institute of Geography Friedrich‐Alexander University Erlangen‐Nuremberg Erlangen Germany; ^5^ Department of Forestry and Environmental Science Shahjalal University of Science and Technology Sylhet Bangladesh; ^6^ Delta Areas and Resources Van Hall Larenstein University of Applied Sciences Leeuwarden The Netherlands; ^7^ Wildlife Ecology and Conservation Group Wageningen University Wageningen The Netherlands

**Keywords:** climate–growth relations, CO_2_ effect, CO_2_ fertilization, CO_2_ × climate interactions, dendrochronology, *Toona ciliata*, tropical forest canopy, tropical tree

## Abstract

Atmospheric CO_2_ (*c*_a_) rise changes the physiology and possibly growth of tropical trees, but these effects are likely modified by climate. Such *c*_a_ × climate interactions importantly drive CO_2_ fertilization effects of tropical forests predicted by global vegetation models, but have not been tested empirically. Here we use tree‐ring analyses to quantify how *c*_a_ rise has shifted the sensitivity of tree stem growth to annual fluctuations in rainfall and temperature. We hypothesized that *c*_a_ rise reduces drought sensitivity and increases temperature sensitivity of growth, by reducing transpiration and increasing leaf temperature. These responses were expected for cooler sites. At warmer sites, *c*_a_ rise may cause leaf temperatures to frequently exceed the optimum for photosynthesis, and thus induce increased drought sensitivity and stronger negative effects of temperature. We tested these hypotheses using measurements of 5,318 annual rings from 129 trees of the widely distributed (sub‐)tropical tree species, *Toona ciliata*. We studied growth responses during 1950–2014, a period during which *c*_a_ rose by 28%. Tree‐ring data were obtained from two cooler (mean annual temperature: 20.5–20.7°C) and two warmer (23.5–24.8°C) sites. We tested *c*_a_ × climate interactions, using mixed‐effect models of ring‐width measurements. Our statistical models revealed several significant and robust *c*_a_ × climate interactions. At cooler sites (and seasons), *c*_a_ × climate interactions showed good agreement with hypothesized growth responses of reduced drought sensitivity and increased temperature sensitivity. At warmer sites, drought sensitivity increased with increasing *c*_a_, as predicted, and hot years caused stronger growth reduction at high *c*_a_. Overall, *c*_a_ rise has significantly modified sensitivity of *Toona* stem growth to climatic variation, but these changes depended on mean climate. Our study suggests that effects of *c*_a_ rise on tropical tree growth may be more complex and less stimulatory than commonly assumed and require a better representation in global vegetation models.

## Graphical abstract

Effects of rising atmospheric CO_2_ levels (*c*_a_) on tree growth are expected to be modified by climate. Using tree‐ring analysis, we found support for hypothesized interactive effects on tropical tree growth for cooler (1a, 2b) and warmer (1b, 2a) sites. Thus, recent *c*_a_ rise has modified growth sensitivity to climatic variation, but the direction depends on mean climate.

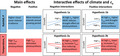

## 1 INTRODUCTION

Tropical forests account for a third of global gross and net primary productivity, store 25% of the carbon in terrestrial ecosystems (Beer et al., 2010; Bonan, 2008) and drive fluctuations of the land carbon sink (Friedlingstein et al., 2019). Their responses to future atmospheric CO_2_ (*c*_a_) rise and warming will thus influence the pace of climate change (Mitchard, 2018). A key challenge in model predictions of tropical forest responses to these changes is the uncertainty of the magnitude of effects of elevated CO_2_ levels, commonly referred to as ‘CO_2_ fertilization effects’ (Cernusak et al., 2013; Fatichi, Pappas, Zscheischler, & Leuzinger, 2019; Körner, 2009; Lewis, Edwards, & Galbraith, 2015; Settele et al., 2014; Terrer et al., 2019). Rise of *c*_a_ increases photosynthetic efficiency (Lloyd & Farquhar, 2008), reduces water use (Cernusak et al., 2013) and may thus stimulate tropical tree growth (Cernusak et al., 2013). These effects can mitigate negative impact of warming on tropical forest productivity, as predicted by dynamic global vegetation models (DGVMs; Cox et al., 2013; Huntingford et al., 2013). Yet, most DGVMs likely overestimate effects of CO_2_ rise as they do not include nutrient or hydraulic limitations (Fatichi et al., 2019; Körner, 2009; Smith et al., 2014; Yang, Thornton, Ricciuto, & Hoffman, 2016). Model benchmarking using empirical studies on *c*_a_‐rise effects is therefore needed (Clark et al., 2017; Zuidema, Poulter, & Frank, 2018).

The effects of *c*_a_ rise on tropical tree photosynthesis and water use are modified by rainfall and temperature (Cernusak et al., 2013; Körner, 2009; Lloyd & Farquhar, 2008) and may thus vary along climatic gradients or with temporal climatic fluctuations. Such *c*_a_ × climate interactions likely drive biome‐specific *c*_a_ responses (Baig, Medlyn, Mercado, & Zaehle, 2015; Hickler et al., 2008; Norby et al., 2016). Reduced drought sensitivity under *c*_a_ rise may be particularly important in regions with low precipitation (P) and during dry years (Cernusak et al., 2013; Fatichi et al., 2016; Zuidema et al., 2013). The effect of *c*_a_ rise on photosynthesis is likely also modified by temperature (*T*). A positive effect of *c*_a_ rise on photosynthetic efficiency can be stronger in warmer regions or during warmer years, as photorespiration increases with temperature and stronger *c*_a_‐driven reduction in photorespiration is therefore expected at high temperature (Cernusak et al., 2013; Long, 1991). On the other hand, *c*_a_ rise may also lead to growth reduction at high temperatures if stomatal closure reduces transpiration, leading to leaf warming beyond the optimum temperature for photosynthesis (Cernusak et al., 2013; Wood, Cavaleri, & Reed, 2012).

Studies on *c*_a_ × climate interactions of (sub‐)tropical trees have been limited to seedling experiments under controlled conditions (Cernusak et al., 2011; Fauset et al., 2019; Kelly, Duursma, Atwell, Tissue, & Medlyn, 2016; de Oliveira & Marenco, 2019a, 2019b; Quentin, Barton, Crous, & Ellsworth, 2015). Responses of canopy trees may be different, as these experience higher irradiance, temperature and vapour pressure deficit. Understanding tropical canopy tree responses to *c*_a_ × climate interactions is crucial as they are responsible for the main share of photosynthesis and biomass in tropical forests (Slik et al., 2013) and are particularly sensitive to drought (Phillips et al., 2009). Tree‐ring analysis represents a powerful method to evaluate effects of *c*_a_ rise on canopy tree physiology and growth (Zuidema et al., 2013). So far, tropical tree‐ring studies have consistently shown positive trends in intrinsic water‐use efficiency (iWUE) over the past century (Hietz, Wanek, & Dünisch, 2005; Loader et al., 2011; Nock et al., 2011; Rahman, Islam, Gebrekirstos, & Bräuning, 2019; van der Sleen et al., 2015), but no consistent growth stimulation (Groenendijk et al., 2015; Nock et al., 2011; Rahman et al., 2019; van der Sleen et al., 2015). These studies were conducted using trend analyses of growth or iWUE. While providing important information on changes in tree physiology and growth, such trend analyses cannot detect interactive effects of *c*_a_ and climate on tree growth. Annual tree growth from dated tree rings allows evaluating modifications of sensitivity of tree growth to climate variation by *c*_a_ rise. Such analyses can be considered as an observational version of *c*_a_ × climate experiments in climate chambers, open‐top chambers or Free Air CO_2_ Enrichment experiments and may also be used to benchmark DGVMs (Baig et al., 2015). To our knowledge, tree‐ring‐based analyses of *c*_a_ × climate interactions are very scarce (Voelker et al., 2017; Wyckoff & Bowers, 2010) and have not been conducted for any tropical tree species.

**FIGURE 1:**
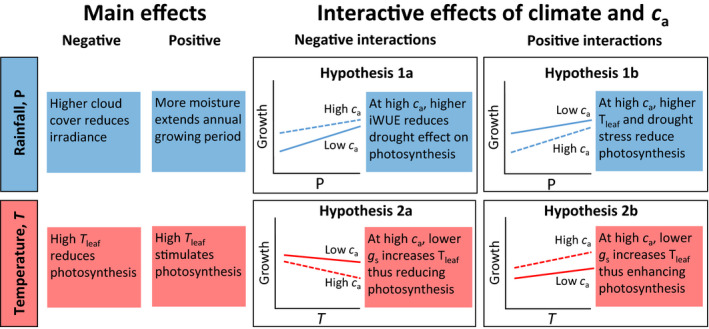
Hypothesized interactive effects of *c*_a_ with rainfall and temperature on tropical tree growth. Four Hypotheses (1a, 1b, 2a, 2b) on *c*_a_ × climate interactions are tested in this study. The graphs show hypothesized interactions as shifts in slope: if the high‐*c*_a_ slope is smaller than the low‐*c*_a_ slope, the interaction is negative; if the reverse is found the interaction is positive. Thus, the vertical position or the direction of the main effect is not relevant for the sign of the interaction. The direction of main and interactive effects depends on mean climate. For main effects, negative P effects are expected at wet sites; positive effects at drier sites; negative T effects are expected at warm sites and positive at cooler site. For interactive effects, Hypotheses 1a and 2b are expected at cooler sites; Hypotheses 1b and 2a at warmer sites. *T*_leaf_ = is leaf temperature; *g*_s_ = stomatal conductance; iWUE = intrinsic water‐use efficiency

Here we use tree‐ring‐width chronologies for a long‐lived tropical tree species (*Toona ciliata*) from four sites with contrasting climate, to evaluate *c*_a_ × climate interactions. Mean climate at these sites allowed comparing *c*_a_ × climate interactions at cooler and warmer sites. In Figure 1, hypothesized interactions are represented by differences in slopes: if the slope of a relation between growth and climate is steeper for high *c*_a_ than low *c*_a_, the interaction is positive; if the slope is less steep at high *c*_a_, the interaction is negative. We tested two alternative hypotheses on *c*_a_ × P interactions (1a and 1b; Figure 1) and two on *c*_a_ × *T* interactions (2a and 2b; Figure 1):

Hypothesis 1a At higher *c*_a_, the growth reduction during dry years is smaller than at lower *c*_a_, because of a higher iWUE. In climate–growth analyses of tree‐ring chronologies, this would lead to reduced sensitivity of ring width to rainfall, causing the positive slope of the growth–rainfall relation to become flatter at high *c*_a_. This is represented by a negative *c*_a_ × P interaction.

Hypothesis 1b The negative *c*_a_ × P interaction of Hypothesis 1a may shift to a positive interaction at warm sites. At these sites, the negative effect of low rainfall on photosynthesis may be stronger at high *c*_a_ because stomatal closure (due to both *c*_a_ rise and drought) causes leaf warming beyond optimal temperature for photosynthesis. Thus, at warm sites, the positive growth–rainfall relation may become steeper at high *c*_a_. This is represented by a positive *c*_a_ × P interaction.

Hypothesis 2a At warm sites, *c*_a_ rise is expected to enhance the negative effects of temperature on photosynthesis as stomatal conductance is reduced, which increases leaf temperature and thus increases the time that leaf temperature exceeds optimal temperature for photosynthesis (Cernusak et al., 2013; Lloyd & Farquhar, 2008). At these sites, the commonly found negative growth–temperature relation may therefore become more negative at high *c*_a_. This would result in negative *c*_a_ × T interactions, particularly for *T*_max_.

Hypothesis 2b At cooler sites, *c*_a_ rise could enhance the positive effect of temperature on photosynthesis (Lloyd & Farquhar, 2008) and growth (Voelker et al., 2017), as *c*_a_‐induced additional leaf warming increases photosynthesis. This is represented by a positive *c*_a_ × T interaction.

Our statistical analysis of *c*_a_ × climate interactions of tree‐ring width in *T. ciliata* revealed that recent *c*_a_ rise caused a significant change in the sensitivity of growth to climatic variation. These *c*_a_ × climate interactive effects varied across sites that differ in mean climate, providing support for Hypotheses 1a and 2b at cooler sites (2b: during the cooler dry seasons), and Hypotheses 1b and 2a at warmer sites.

## 2 MATERIALS AND METHODS

### 2.1 Study species

**FIGURE 2:**
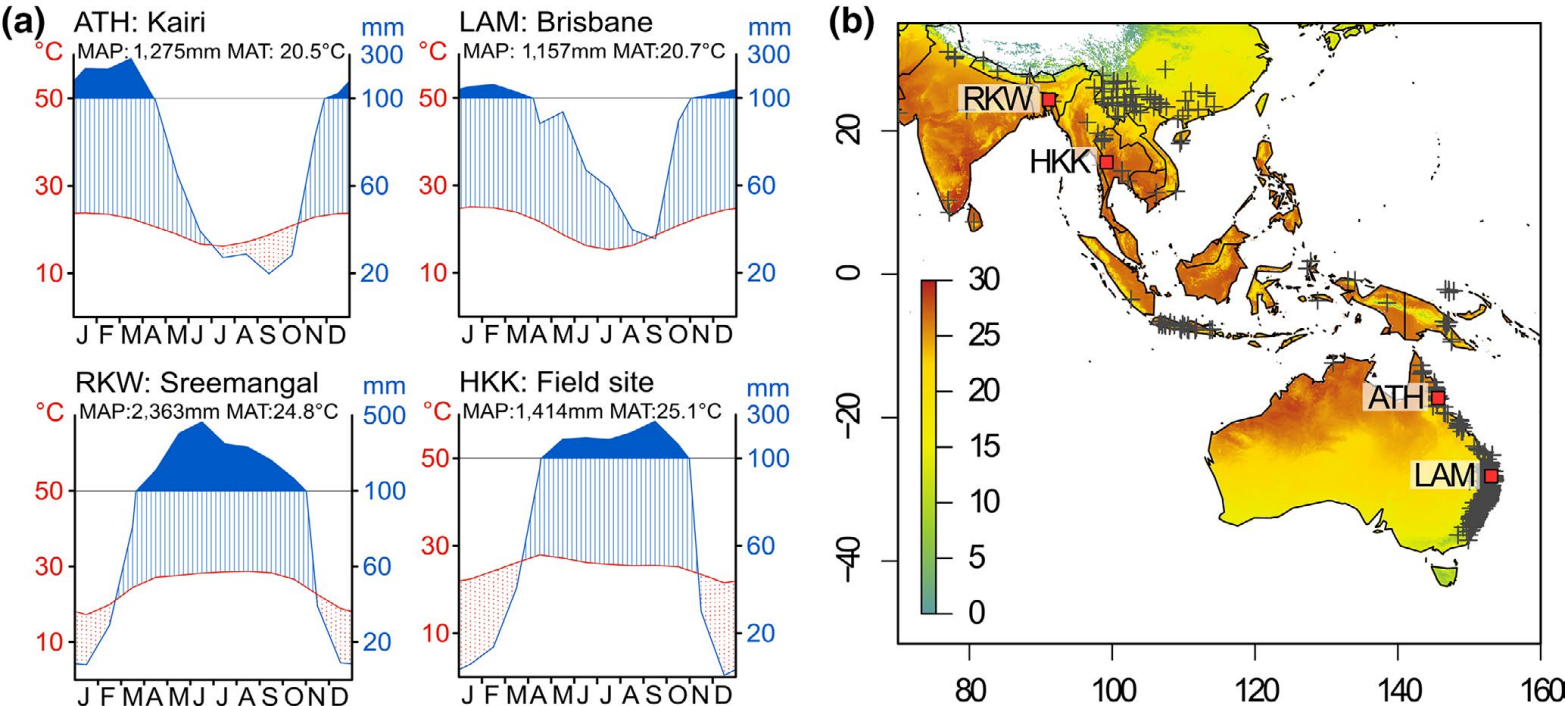
Climatic characteristics and location of our study sites. (a) Climate diagrams for the study sites, from nearby climate stations (ATH, LAM and RKW) or based on gridded data (HKK). (b) Locations of the four study sites on a map showing mean annual temperature (°C) based on WorldClim 2.0 gridded data (Fick & Hijmans, 2017). Crosses indicate locations where *Toona ciliata* herbarium specimens were collected as an indication of the natural geographic range (GBIF.org, 2019)

We studied *T. ciliata* (Meliaceae), a large‐stature and long‐lived pioneer tree species distributed in South Asia, South‐East Asia and Australia (Figure 2b). *T. ciliata* is usually deciduous or semi‐deciduous but under optimal growing conditions saplings can show opportunistic non‐deciduous behaviour (Heinrich & Banks, 2006). This species is ideally suited to study *c*_a_ × climate interactions as it forms growth rings that are reliably dated (Heinrich, Weidner, Helle, Vos, & Banks, 2008; Rahman, Islam, & Islam, 2017; Vlam, Baker, Bunyavejchewin, & Zuidema, 2014), produces high‐quality growth chronologies, exhibits clear climate–growth relations (Heinrich et al., 2008, 2009; Vlam et al., 2014), reaches >150 years in age (Heinrich et al., 2008; Vlam, van der Sleen, Groenendijk, & Zuidema, 2017) and occurs across a large climatic range (Figure S1). *T. ciliata* forms distinct (semi) ring‐porous growth rings, characterized by initial parenchyma and large early‐wood vessels (Heinrich & Banks, 2005; Islam, Rahman, & Bräuning, 2018).

*T. ciliata* is distributed in seasonally dry tropical forests, which represent half of all tropical forests (Guan et al., 2015) and likely respond more strongly to *c*_a_ rise than wetter forests (Cernusak et al., 2013; Fatichi et al., 2016; Norby et al., 2016). Across its range, the species is distinctly deciduous, with leafless periods of typically several weeks (Rahman et al., 2017; Vlam et al., 2014) to several months (Heinrich & Banks, 2005).

### 2.2 Study sites and climate data

We used tree‐ring‐width measurements from published chronologies of *T. ciliata*. Tree‐ring data were available from four sites in Australia and South and South‐East Asia which vary in rainfall and particularly in temperature (Table 1; Figure 2a). Characteristics of the original chronologies are included in Table S1 and show a high dating accuracy and a strong common response to environmental drivers at all sites. All sites are located in nature reserves with no signs of logging or silviculture. Annual rainfall distribution at all sites is unimodal (Figure 2a), with peaks during northern hemisphere (SE Asian sites) or austral summer (Australian sites). Annual temperature also fluctuates during the year: wet seasons are warmer and dry seasons cooler (Figure 2a).

**TABLE 1:** Study sites and site‐specific characteristics of *Toona ciliata*

Site	Name	Country	Study period	Wet season	Leafless	*N*
ATH	Atherton	Australia	1950–1998	Dec–Mar	Jul–Aug	37
LAM	Lamington	Australia	1950–1999	Nov–Mar	Jun–Aug	20
RKW	Rema‐Kalenga	Bangladesh	1951–2014	Apr–Oct	Jan–mid Feb	26
HKK	Huai Kha Khaeng	Thailand	1953–2010	May–Oct	0.5 months	46

Note

Sites are ordered with increasing mean annual temperature. ‘Leafless’: period during which trees were observed to be deciduous; ‘Wet season’: months with >100 mm rainfall; *N*: number of sampled trees. Original publications: ATH (Heinrich et al., 2008), LAM (Heinrich et al., 2009), HKK (Vlam et al., 2014) and RKW (Rahman et al., 2018).

Climate data (monthly *T*_max_ and precipitation) were obtained from nearby climate stations (Figure 2; Table S1). For the ATH site, station data for *T*_max_ were unavailable prior to 1967; we therefore obtained *T*_max_ from gridded CRU data (Harris, Jones, Osborn, & Lister, 2014) and rainfall from station data. For the HKK site, long‐term climate data were available only from Nahkon Sawan (Figure S2), which is located >85 km away, at a 500 m lower elevation, experiencing a 5°C higher temperature than recorded on site (Bunyavejchewin, 2009). To characterize this site in terms of climate, we therefore used gridded WorldClim data (Fick & Hijmans, 2017). Gridded average temperature and rainfall (Table 1) are close to those recorded at the site (MAT site: 23.5°C; MAP site: 1,473 mm) during a 6‐year period (Bunyavejchewin, 2009). For our climate–growth analyses, Nahkon Sawan station data could be used as all climate data are scaled prior to analyses and absolute temperature or rainfall differences do therefore not matter.

Data on *c*_a_ were obtained from the Mauna Loa atmospheric CO_2_ records (www.esrl.noaa.gov/gmd/ccgg/trends). Per site, the study period was determined by the overlapping period for which tree‐ring data (Table S1) and climate data were available (Table 1). Study periods varied from 49 to 64 years, all within 1950–2014 and with a common period of 1953–1998. During the periods covered by the chronologies, *c*_a_ increased by 18%–28% for individual chronologies, by 28% for the total period and by 17% for the common period.

As climate × *c*_a_ interactions may vary between wet and dry seasons, we performed our analyses using seasonal climate data. For each site, we defined the wet season as the period composed of months with >100 mm precipitation on average during the study period. This resulted in wet seasons’ lengths of 4–7 months. Above 100 mm/month, water limitation is considered to be small for lowland tropical trees, and this cut‐off has been used in previous studies on climate–growth relations of *T. ciliata* (Vlam et al., 2014). As *T. ciliata* is deciduous for only <1–2 months at our study sites, climatic conditions during parts of the dry season may also drive annual tree growth. To evaluate interactive climate × *c*_a_ effects during these transitional months, we grouped the 2 months prior and following the wet season and included these transitional months as the ‘Dry season’ in our analysis. We do recognize that this is not a true season, but retained this term for ease of communication. There were three reasons to implement dry‐season rainfall in this way. First, combining climatic data for these transitional months into one dry‐season value reduces the number of explanatory variables in our statistical models, and thus avoids overparameterization and co‐linearity. Second, we do not expect effects of rainfall or temperature during the driest months when our study species is commonly leafless. Third, published climate–growth relations at monthly scales for our study sites and species show that climate effects occur both in wet season and transitional months (Heinrich et al., 2008, 2009; Rahman, Islam, & Bräuning, 2018; Vlam et al., 2014), in accordance with meta‐analyses of climate–growth relations in tropical trees (Rozendaal & Zuidema, 2011).

For all sites, we tested for linear trends in climate data within the study periods. Results of these tests revealed a significant (*p* < .05) increase in *T*_max_ at the HKK site, in both wet and dry seasons (Figure S3), but not in any of the other sites. No significant trends in rainfall were found at any site. We also tested for trends in *T*_min_ (not included in our statistical analyses) and found significant increases for all sites except LAM.

Per site we calculated the number of days in the wet and dry season during which *T*_max_ exceeded 30°C. At this temperature, canopy tree leaves likely reach temperatures of >32°C (Pau, Detto, Kim, & Still, 2018) which is above optimal for photosynthesis (Mau, Reed, Wood, & Cavaleri, 2018; Pau et al., 2018). This analysis revealed that the incidence of such hot days was higher at warmer sites (particularly during the dry season) and also increased over time at these sites (Figure S4).

### 2.3 Data collection and tree‐ring measurements

At all sites, increment cores were collected from natural populations of *T. ciliata* in extensive forest areas (>500 ha in all cases). At ATH and LAM sites, sampled trees were selected to be sub‐dominant or dominant, with 30–40 m (ATH) or 40–50 m (LAM) in height and 80–120 (ATH) or 80–300 cm (LAM) in diameter at breast height (DBH). At HKK and RKW, trees of >5 cm DBH were selected and maximum DBH was 115 and 25 cm DBH, respectively. Coring height was 1.0 m at HKK and 1.3 m at other sites. While the selection of tree sizes differed between sites, chronology statistics were similar (Table S1). A total of 20–46 trees were sampled per site, with 1–2 (ATH, LAM and RKW) or 2–3 cores per tree (HKK). Sample preparation, scanning, microscopy, ring measurements and cross‐dating followed standard and fully exchangeable dendrochronological practices, resulting in ring‐width measurements to the nearest 0.01 mm. Dating of rings at the two Australian sites followed the Schulman convention (Schulman, 1956), implying that the ring is dated according to the year in which ring formation starts. So, a growth ring at the ATH site that started to be formed in end of 1980 (start of austral summer) is dated ‘1980’, but continues into 1981 and is influenced by climatic conditions during both 1980 and 1981: wet season (December 1980–March 1981) and dry season (October–November 1980 and April–May 1981).

The raw ring‐width measurements of trees that were part of the chronology are the basis for the analyses in this study. By using only these trees, we have a high certainty that only correctly dated individual trees are included in the analyses. A total of 5,318 ring‐width measurements were included in our analyses.

### 2.4 Statistical analyses

We evaluated the significance and magnitude of *c*_a_ × climate interactions on *T. ciliata* diameter growth using mixed‐effect models (MEMs; Zuur, Ieno, Walker, Saveliev, and Smith (2009)). We applied MEMs instead of more commonly used correlation analyses in tree‐ring research as MEMs allow (a) testing for effects of multiple and interactive explanatory variables of ring‐width variation at the same time, (b) accounting for the repeated measurement structure of the data (by including tree individual as a random factor) and (c) quantifying the degree to which variation in ring width is explained by intrinsic (within individual) and extrinsic (environmental factors and their interactions) sources of growth variation. MEMs have recently been used to study climate–growth relations of tree‐ring data (Galván, Camarero, & Gutiérrez, 2014; Gea‐Izquierdo, Cherubini, & Cañellas, 2011), also for our study species (Vlam et al., 2014). For all sites, tree‐ring width was log‐transformed to obtain a normal distribution of residuals.

We chose not to detrend ring‐width series, as this may affect the detection of climate–*c*_a_ interactions. Instead, we accounted for ontogenetic effects on ring width by adding tree age at ring formation (‘Age’) as explanatory variable in the MEM. Age was estimated as cambial age for HKK (Vlam et al., 2014) or estimated by the number of rings measured in each series, for the other sites. The former accounts for missing distance to the pith; the latter does not. Means of these age estimates were 80, 84, 46 and 58 years for ATH, LAM, RKW and HKK, respectively; maximum tree ages were 185, 144, 83 and 150 years. We also tested whether adding age^2^ to MEMs would improve model fit or alter model output, but this was not the case. Note that non‐linear ontogenetic effects on growth were also accounted for by log transformation of ring width.

We accounted for growth heterogeneity within the population by adding TreeID as random variable (random intercept) in the MEM. We did not include TreeID as random slope because we did not aim to account for individual variation in climate–growth relations and as this would involve adding a large number of random slopes which results in highly complex models. We checked whether adding an autocorrelation structure to account for temporal autocorrelation in tree growth (Vlam et al., 2014) would change the MEM output. This was not the case, and we therefore did not include autocorrelation in the analyses presented here.

Four groups of explanatory variables were included in our MEMs: (a) *T*_max_ and P, for both the wet season and dry season; (b) *c*_a_; (c) interactions of *c*_a_ and season‐specific *T*_max_ and P and (d) age. All explanatory variables were scaled (mean = 0, *SD* = 1) prior to inclusion in MEMs, to obtain standardized coefficients and allow for direct comparison of the strength of effects. For each MEM, we evaluated collinearity of the explanatory seasonal climate variables; we did not find cases for which the variance inflation factor exceeded 5.

Per site, we constructed three sets of MEMs. Set A: to evaluate general sensitivity of *Toona* growth to climate, we performed a climate–growth analyses using only climate and age variables (groups a and d). Set B: to test Hypotheses 1a, 1b, 2a, 2b of *c*_a_ × climate interactions, we ran models including all variables (groups a–d), with age to account for ontogenetic effects on growth. A comparison of the model output of sets A and B allowed us to evaluate the extent to which climate–growth relations are modified by *c*_a_ × climate interactions. Set C: as set B, but for a common period of 1953–1998 to verify robustness of model output to differences in duration and timing of study periods among the study sites.

We ran one MEM per site and did not combine sites into one MEM, in order to prevent modelling three‐way interactions. We used backwards selection of explanatory variables, based on AIC change (ΔAIC ≥ 2), taking the simplest model in case of ΔAIC < 2. Analyses were performed using the lme function of the nlme package in R (R Core Team, 2019). We calculated conditional and marginal *R* ^2^ to evaluate variation explained by fixed effects alone and fixed and random effects together (MuMIn package).

## 3 RESULTS

### 3.1 Accounting for ontogeny and individual heterogeneity

**FIGURE 3:**
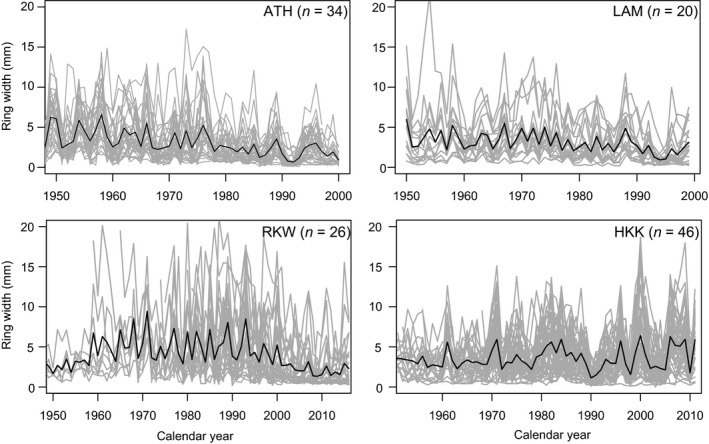
Raw ring‐width series of *Toona ciliata* trees (grey) and their mean (black) at four study sites. Sites abbreviations are explained in Table 1; sites are ordered with increasing mean annual temperature. The mean chronology (without detrending) is only shown for illustration purposes; all statistical analyses were performed on ring‐width series for individual trees

Ring‐width data for all four sites show strong annual variation in growth of individual trees, with a considerable degree of common growth variation (Figure 3). The clear and synchronous occurrence of narrow and wide rings in these raw ring‐width series illustrate that our study species are climate sensitive. The raw ring‐width data also show that individual trees differ widely in growth.

Effects of age on ring width were significant and negative for all sites and for all model sets. These ontogenetic effects on (log‐transformed) ring‐width are illustrated in Figure S5.

### 3.2 Growth sensitivity to climatic variation

The set‐A mixed‐effects models, in which effects of climatic variation on ring width were tested (Table 2), showed positive effects of dry‐season rainfall on growth at two sites (ATH and HKK) and negative wet‐season rainfall effects at one site (HKK). The latter is possibly associated with increased cloud cover during wet years.

**TABLE 2:** Results of three sets of mixed‐effect models (A–C) evaluating effects of climate and *c*_a_ × climate interactions on ring width of *Toona ciliata* at four study sites

Site	Model set	AIC	*R* ^2^_m_	*R* ^2^_c_	*df*	*N*
ATH	A. Climate effects only	3,662	.22	.35	1,541	1,580
	B. With climate × *c*_a_ interactions	3,635	.25	.38	1,537	1,580
	C. As B, but for common period	3,416	.25	.38	1,441	1,482
LAM	A. Climate effects only	1,903	.16	.45	915	939
	B. With climate × *c*_a_ interactions	1,886	.18	.47	911	939
	C. As B, but for common period	1,738	.20	.48	846	874
RKW	A. Climate effects only	2,646	.25	.37	1,051	1,079
	B. With climate × *c*_a_ interactions	2,647	.26	.37	1,050	1,079
	C. As B, but for common period	124	.12	.22	663	692
HKK	A. Climate effects only	5,327	.11	.25	2,237	2,289
	B. With climate × *c*_a_ interactions	5,299	.13	.27	2,235	2,289
	C. As B, but for common period	3,926	.11	.28	1,686	1,738

Note

**FIGURE 4:**
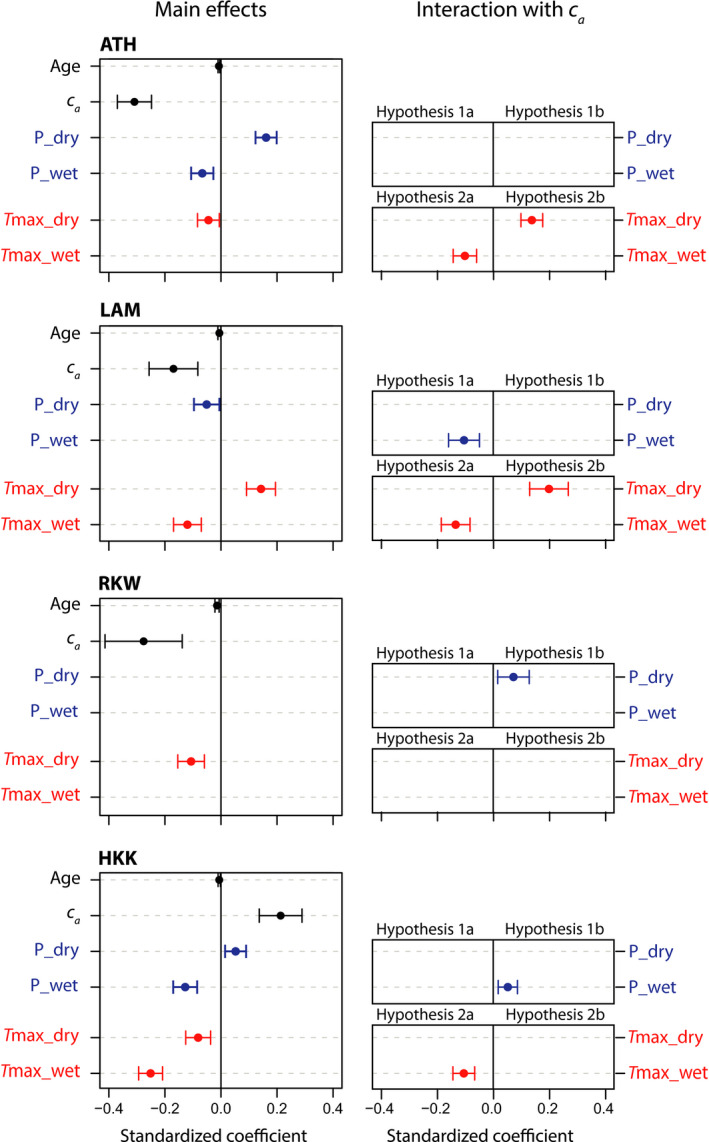
Mixed‐effect model results of *c*_a_ × climate effects on *Toona ciliata* ring width, at four study sites. Shown are standardized coefficients (mean and 95% confidence interval) of main climate effects and *c*_a_ × climate interactions (Hypotheses 1a, 1b, 2a, 2b). Tested seasonal climate variables (wet and dry) are precipitation (P, blue) and maximum temperature (*T*_max_, red). Sites ATH and LAM are cooler; RKW and HKK are warmer

Sites ATH and LAM are cooler; RKW and HKK are warmer. For the selected models, the table includes AIC, marginal *R* ^2^ (*R* ^2^_m_, based on fixed effects only), conditional *R* ^2^ (*R* ^2^_c_, both fixed and random effects), degrees of freedom (*df*) and sample size (*N*). Coefficients are shown in Figure S6 (set A), Figure 4 (set B) and Figure S7 (set C).

Effects of temperature differed between warmer (HKK and RKW) and cooler sites (ATH and LAM; Figure S3). At warmer sites, years with higher seasonal temperature (*T*_max_) reduced ring width in three out of four season × temperature combinations, and did not lead to growth stimulation in any case (i.e. we did not find positive coefficients for temperature). These negative effects are consistent with the expected effects of high air temperature on photosynthesis (through high *T*_leaf_), water stress and respiration (Figure 1). Analyses of daily climate values for these sites revealed that *T*_max_ exceeded 30°C on up to 142 of wet‐season days and up to 71 of dry‐season days (Figure S4), likely leading to temperature‐induced reductions of photosynthesis. At one of the cooler sites (LAM), a positive effect of temperature (*T*_max_) was found during the dry season, when temperatures are lower (Figure 2). Higher temperatures during these dry and cool months may stimulate photosynthesis and wood formation.

### 3.3 Climate × *c*_a_ interactions in MEMs

The effect of *c*_a_ on the climate–growth relations of *T. ciliata* can be inferred by comparing results of MEM sets A and B (without or with *c*_a_ × climate interactions). First, at three of the four sites, adding *c*_a_ × climate interactions improved model fit and increased variance explained by fixed variables (*R* ^2^_m_; Table 2). Thus, adding *c*_a_ × climate interactions increased explained variation in annual ring width of our study species. Yet, change in *R* ^2^_m_ was small (0.01–0.03; Table 2), which indicates a reorganization of explained variance rather than the explanation of additional variance.

Second, inclusion of *c*_a_ × climate interactions caused shifts in the sets of climate effects that were significant (compare Figure S6 and main effects in Figure 4). While the sign of significant coefficients never shifted between sets A and B, significant climate effects in set A sometimes shifted to non‐significant in set B, and vice versa. For instance, at the ATH site, the model including interactions (Figure 4) contained significant negative effects of *T*_max_ during the dry season and rainfall during the wet season, which were absent from the model without interactions (Figure S6).

Third, a comparison of the standardized coefficients of climate × *c*_a_ interactions and main climate effects show that these have a comparable magnitude. The average of absolute coefficients of interactions equalled 0.12 (*SD* = 0.09), while that for main effects was 0.11 (*SD* 0.04). Overall, these comparisons show that adding *c*_a_ in climate–growth analyses for *Toona* improved model fit, slightly increased explained variance, caused shifts in significant climatic drivers and yielded interactive effects that have comparable magnitude to main climate effects.

### 3.4 Rainfall × *c*_a_ interactions

Rainfall × *c*_a_ interactions in MEMs (set B) differed between sites (Figure 4). At one of the two cooler sites (LAM), we found a negative interactions for wet‐season precipitation. This is consistent with Hypothesis 1a: the expected response of a *c*_a_‐induced reduction in drought sensitivity. At the two warmer sites (HKK and RKW), we found positive rainfall × *c*_a_ interactions. This implies that positive slopes of the ring‐width versus rainfall relations became steeper at higher *c*_a_ levels or negative slopes became less negative. These interactions are in accordance with Hypothesis 1b and are expected at warmer sites. At these sites, the *c*_a_‐induced reduction of *g*_s_ may cause *T*_leaf_ during dry years to frequently exceed optimum temperature for photosynthesis. This effect may be enhanced if dry years also tend to be warm years, which was the case for both warmer sites (negative correlations between *T*_max_ and P, Table S2). In addition, these two sites also experienced increase in *T*_min_ over time (Figure S3), which may counteract the reduction in drought sensitivity occurring with gradual *c*_a_ rise. In our MEMs, such warming‐induced change in drought sensitivity could become apparent as positive *c*_a_ × rainfall interactions, even if the mechanism is warming. Thus, increase in seasonal *T*_max_ and *T*_min_ during the study period at RKW and HKK may have induced an increase in drought sensitivity that was stronger than the reduction in drought sensitivity induced by *c*_a_ rise.

Analyses for the common period (Set C, Table 2; Figure S7) yielded the same *c*_a_ × rainfall interactions for two sites (ATH and LAM), a seasonal shift at one site (RKW) and loss of a temperature interaction at the HKK site. Thus, overall *c*_a_ × temperature interactions were quite robust to the shift in period.

### 3.5 Temperature × *c*_a_ interactions

Temperature × *c*_a_ interactions in MEMs (set B) also differed between sites (Figure 4). We expected positive interactions at cooler sites (LAM & ATH) because of the stronger reduction in photorespiration during warm years and because at these sites of *T*_leaf_ does not often exceed the optimum temperature for photosynthesis. The positive interactions of *c*_a_ with *T*_max_ during the dry (=cooler) season at LAM and the positive interaction of *c*_a_ with *T*_min_ in the wet season at ATH are consistent with such a response. Yet, a negative interaction (*T*_max_ in wet season) was also found at LAM, which is not expected for a relatively cool site where *T*_max_ during the wet season averages 25°C. The negative correlation between wet‐season *T*_max_ and rainfall may help explaining these interactions: hotter wet seasons also tend to be drier (Table S2). Another possible explanation is that the incidence of days during which *T*_max_ leads to leaf temperatures exceeding optimum for photosynthesis is similar for cooler and warmer sites during the wet season. This seems to be the case for the LAM site (Figure S4).

At the two warmer sites, one negative interaction was found (HKK), in accordance with Hypothesis 2a. This may be explained by the *c*_a_‐induced reduction in *g*_s_ and increase in *T*_leaf_, and is supported by the high frequency of hot days (*T*_max_ > 30°C; Figure S4). Yet, negative interactions at HKK may also have resulted from the significant warming (Figure S2), which occurred alongside *c*_a_ rise. Effects of *c*_a_ rise and warming cannot be distinguished and warming may have aggravated negative effects of *T*_max_ during the wet season (HKK).

Models for the common period (set C) yielded the same *c*_a_ × *T*_max_ interactions for the cooler sites, a seasonal shift at one warmer site (HKK) and a new (positive) interaction at the other warm site (RKW). The latter change is not in accordance with the expected response for a warm site (and during the warmer season), and we do not have an explanation for this result. In general, the change from set B to set C did not cause a major shift in MEM results that could be explained by recent global warming and recent increase in hot years at the warmer sites (Figure S4).

## 4 DISCUSSION

Our analysis of ring‐width measurements of *T. ciliata* revealed that recent *c*_a_ rise caused a significant change in the sensitivity of tree growth to climatic variation. This shift in sensitivity was evidenced by a better fit of statistical models that included *c*_a_ × climate interactions (at three sites), the presence of robust *c*_a_ × climate interactions in these models, and the changes in climate–growth relations between models with and without *c*_a_ × climate interactions. We found that *c*_a_ × climate interactive effects on tree growth varied across sites that differ in mean climate. For the two cooler sites in our dataset, results agree with hypothesized reduction in drought sensitivity (negative interaction, Hypotheses 1a). Temperature sensitivity shifted in different directions: the expected positive effect of *c*_a_ rise on ring width was found during the cooler dry season (Hypothesis 2b), whereas negative interactions were found during the hotter wet season (Hypothesis 2a). For the two warmer sites, drought sensitivity increased under *c*_a_ rise and temperature sensitivity shifted such that hot years caused a stronger growth reduction. Both responses were in accordance with hypotheses for warm sites (Hypotheses 1b and 2a).

### 4.1 Climate × *c*_a_ interactive effects on *Toona* growth

The studied *T. ciliata* trees included in this study grew during a period when *c*_a_ increased by 28%. For one of the sites (HKK), stable isotope analyses revealed that *c*_a_ rise caused an increase in iWUE of approximately 35% for our study species during this period (Nock et al., 2011; van der Sleen et al., 2015). Thus, *Toona* physiology has responded to *c*_a_ rise at that site, and similar responses are likely for the other sites, given the generic *c*_a_‐induced iWUE increases for tropical tree species worldwide (van der Sleen, Zuidema, & Pons, 2017). Increased iWUE response likely involved both a reduction in transpiration (due to a reduction in stomatal conductance) and increased photosynthesis (due to an increase in *c*_i_). Tree‐ring‐based analyses of growth trends for the RKW and HKK sites did not yield evidence that these physiological changes have stimulated growth (Groenendijk et al., 2015; Nock et al., 2011; Rahman et al., 2018; van der Sleen et al., 2015). This suggests that tree growth is more strongly limited by rainfall, heat and/or nutrient availability than by *c*_a_; or that trees respond to *c*_a_ rise by increasing organ turn‐over rates, respiration or investments in other tree parts (van der Sleen et al., 2015). One of these factors—limitation of diameter growth by high temperature and low rainfall—has indeed been observed of *T. ciliata*, both in the analyses included in this study and elsewhere (Heinrich et al., 2008; Rahman et al., 2018; Shah & Mehrotra, 2017; Vlam et al., 2014).

The above insights into growth‐determining factors and *c*_a_ responses lead to the expectation that *c*_a_ rise would modify climate–growth relations of *Toona*, or—put differently—that effects of *c*_a_ rise on *Toona* growth depend on its sensitivity to climatic fluctuations. Our results confirm that this is the case. Interactive effects of *c*_a_ and climate on *Toona* growth seem to reflect a balance of *c*_a_‐induced increase in water‐use efficiency and decrease in drought sensitivity on the one hand, and *c*_a_‐induced increase in leaf temperature beyond the temperature optimum for photosynthesis on the other hand. These results are in line with those from a mechanistic tree‐growth model, parameterized for *T. ciliata* at the HKK site and forced by observed annual temperature and rainfall (Schippers, Sterck, Vlam, & Zuidema, 2015). Simulations of annual wood production using this model yielded temporal fluctuations that showed high correlations with the HKK tree‐ring chronology, but this match did not improve when effects of *c*_a_ rise were simulated.

Overall, our results suggest that for our study species, climate × *c*_a_ interactions lead to complex responses that do not necessarily result in *c*_a_‐induced growth stimulation and depend on season, site and time period considered. As a result, it seems that for our study species *c*_a_ rise did not cause an overall ‘growth bonus’, but rather induced subtle and variable modifications of climate–growth relations (cf. Clark, Clark, & Oberbauer, 2013).

We deliberately did not interpret the main *c*_a_ effects in our MEMs because tree age and *c*_a_ both increased over time, and hence it is difficult to partition variance driven by ontogeny or by *c*_a_ in our models. Furthermore, we aimed to evaluate the effects of *c*_a_ rise on climate–growth relations, and not on growth averaged over multiple years. We therefore did not conduct any detrending on the tree‐ring series and we assumed a linear effect of age on log‐transformed ring width (confirmed by significant age effects in models and clear ontogenetic relations, Figure 4; Figure S5). Thus, negative or positive effects of *c*_a_ in our statistical models cannot be separated from those caused by ontogenetic effects and climatic trends.

Can similar results be expected for other tropical forest species? *T. ciliata* exhibits climate–growth relations that are similar to those obtained from other tropical forest species (Rozendaal & Zuidema, 2011), has experienced comparable increases in iWUE compared to other tropical species (Nock et al., 2011; van der Sleen et al., 2015) and occurs along large climatic gradients (Figure S1). We therefore expect that studies on other tropical tree species would reveal *c*_a_ × climate interactions that depend on mean climate in similar ways. The strength and abundance of these interactions may differ depending on climate envelops. For instance, *c*_a_ × T interactions would probably be less common for specialists of warmer areas that possess a higher optimal leaf temperature for photosynthesis, and more common for specialists of cooler areas. Clearly, it is important to test this expectation and extend this analysis to other species. Possible extensions are to include other *Toona* species (e.g. *T. sinensis*, *T. fargesii*, *T. sureni* and *T. calantas*) or setting up networks on other tropical tree genera with high potential for tree‐ring analysis (e.g. *Cedrela* and *Entandophragma*). Such networks combine a large climatic variation with limited phylogenetically induced differences in climate responses.

### 4.2 Results of experimental *c*_a_ × climate manipulations

To put our results on *c*_a_ × climate interactive effects on tree growth in context, we performed a qualitative review of *c*_a_ × water and *c*_a_ × T experiments. We focused our review on (sub‐)tropical species, implying that it is not complete for extra‐tropical studies. Our review only included studies on seedlings or small trees, as *c*_a_ × climate experiments for canopy trees or forests are absent. We included 12 publications, in which six tropical, two sub‐tropical and four temperate tree species were studied (Table S3). All studies were conducted in a greenhouse except for one conducted in climate controlled field chambers (Quentin et al., 2015). A total of 19 species × study combinations were included (six *c*_a_ × water studies, seven *c*_a_ × T factorial treatments and six three‐way factorial experiments). For each study, we evaluated whether results are consistent with one of the four hypotheses (Figure 1) and summarized this in Table 3.

**TABLE 3:** Results of a qualitative literature review on tree responses to experimental *c*_a_ × water and *c*_a_ × temperature interactions

Interaction tested	Studies supporting hypothesis			*N*
*c*_a_ × water availability	1a	1b	NS	
	25%	8%	67%	12
*c*_a_ × temperature	2a	2b	NS	
	0%	62%	38%	13

Note

The review included studies from temperate, subtropical and tropical species, see full overview in Table S3. *N* is the number of study × species combinations.

The 12 reviewed *c*_a_ × water experiments yielded variable interactive responses (Table 3). Increased iWUE was found for one tropical species, for which drought‐induced reduction in photosynthesis disappeared under elevated *c*_a_ (de Oliveira & Marenco, 2019a, 2019b). No significant interactions were found for four other tropical species (Cernusak et al., 2011; Kelly et al., 2016). For one subtropical species, the negative effect of drought on photosynthesis was offset by increased *c*_a_ (Hypothesis 1a), whereas the other subtropical species studied did not show significant interactions (Lewis et al., 2013). For temperate species, only one out of four species showed a significant interaction, where photosynthesis increased to a new maximum under high *c*_a_ with increasing water availability (Hypothesis 1b; Duan et al., 2015). Overall, these results show a stronger support for Hypothesis 1a than Hypothesis 1b, although non‐significant results dominate.

The 13 reviewed *c*_a_ × T experiments yielded either positive or non‐significant interactions (Table 3). The only study on a tropical species showed that photosynthetic capacity increased faster with temperature under elevated *c*_a_ (Fauset et al., 2019). For subtropical species, positive *c*_a_ × T interactions were found on plant dry mass and net photosynthesis (Ghannoum et al., 2010; Lewis et al., 2013; Logan et al., 2010). No significant *c*_a_ × T interactions were found for two temperate species. Thus, all significant interactions were in line with Hypothesis 2b (Figure 1). A more comprehensive meta‐analysis of *c*_a_ × T experiments for tree seedlings showed that both positive and negative interactions are found, but overall *c*_a_ × T interaction was non‐significant (Baig et al., 2015).

These experimental results show that under elevated *c*_a_, tree seedlings tend to have a higher temperature sensitivity (enhancing photosynthesis and growth) and a lower drought sensitivity. These responses are consistent with our Hypotheses 1a and 2b (for cooler sites) and with our results for the two cooler sites (*c*_a_ × T for dry season). For the two warmer study sites, our results suggest increasing drought sensitivity under elevated *c*_a_ which is not in accordance with experimental results. This discrepancy is likely explained by the fact that most experimental studies are performed at lower temperatures (and thus leaf temperature) and at lower irradiance levels (and hence air and leaf temperatures) than experienced by the trees included in our study. Thus, in experimental seedlings, leaf temperature likely did not exceed the optimum for photosynthesis, while this appears to be the case for our sampled trees (Figure S4; Mau et al., 2018; Pau et al., 2018).

### 4.3 Using tree rings to detect *c*_a_ × climate interactions in trees

In this study, we applied tree‐ring analyses to explicitly test for climate × *c*_a_ interactions of large trees. This is the first study to do so for tropical trees, and one of the first for trees in general. Two earlier tree‐ring studies on an oak species have evaluated shifts in climate–growth in response to *c*_a_ rise (Voelker et al., 2017; Wyckoff & Bowers, 2010). Voelker et al. (2017) evaluated shifts in temperature sensitivity of oak growth under low and high *c*_a_, by comparing tree‐ring series in paleo and modern wood. They found that temperature sensitivity was stronger in modern oaks that grew under high *c*_a_ (positive interaction, Hypothesis 2b). Wyckoff and Bowers (2010) compared drought sensitivity before and after 1950 and found a reduction in the sensitivity of tree growth to drought (PDSI) with increasing *c*_a_, in line with Hypothesis 1a. Both studies tested for time × climate interactions—rather than *c*_a_ × climate interactions as we did, but in both cases significant interactions were interpreted as effects of *c*_a_ rise. Other tree‐ring studies have also reported significant shifts in climate–growth relations over time, but did not link these to *c*_a_ rise. For instance, a global analysis of tree‐ring studies identified large‐scale shifts in the sensitivity of tree growth to temperature during the 20th century (Babst et al., 2019). The observed decrease in temperature sensitivity in cold‐dry systems (boreal forests) and increased drought sensitivity across temperate and boreal forests are not in accordance with expected responses of *c*_a_ rise for cooler sites (Hypotheses 2b and 1a) and were interpreted to result from warming (Babst et al., 2019). Yet, responses for other regions (cold‐humid and temperate) are consistent with *c*_a_‐induced responses but were not interpreted in this context. Our results and these examples show that *c*_a_ rise may help interpreting recent shifts in climate–growth relations, and we suggest that tree‐ring researchers evaluate this possibility in dendrochronological studies.

What is the potential of using tree‐ring chronologies to evaluate *c*_a_ × climate interactions on tree growth? Important virtues of tree‐ring based analyses include the flexibility in selecting locations, the spatial and temporal extent at which studies can be performed, and the fact that responses of canopy trees are evaluated (instead of seedlings). Species producing annual tree rings are distributed across many biomes and recent advances in tropical dendrochronology now allow such analysis for tropical forests (Brienen, Schöngart, & Zuidema, 2016). A second advantage is the public availability of thousands of tree‐ring chronologies in international databases (e.g. International Tree‐Ring Data Bank [ITRDB]), albeit with limited representation of tropical species (Babst et al., 2019). And finally, the analysis of annual variation in tree growth to evaluate effects of *c*_a_ rise on tree growth avoids methodological issues in growth trend analyses using tree rings (Brienen, Gloor, & Ziv, 2017; van der Sleen, Groenendijk, et al., 2017).

There are also several drawbacks of the use of tree‐ring chronologies to evaluate *c*_a_ × climate interactions. For instance, tree‐ring‐based inferences on *c*_a_ effects are limited to the responses to past *c*_a_ levels, which can differ from those to future *c*_a_ rise. Second, chronologies are often constructed for the most climate‐sensitive populations or individuals, thus overestimating climate effects (Klesse et al., 2018). Third, a large share of chronologies stored in international databases does not include growth rates in recent decades, and is thus of limited use (Babst et al., 2018). And finally, analyses of tree‐ring chronologies often do not allow separating effects of concurrent *c*_a_ rise and warming on tree growth. We therefore call for multifactorial experiments to be conducted on tropical tree species to quantify effects of temperature and *c*_a_, preferably on canopy trees (Cavaleri, Reed, Smith, & Wood, 2015; Zuidema et al., 2013).

### 4.4 Implications and concluding remarks

Overall, we conclude that tree‐ring analyses can provide an important contribution in testing and quantifying *c*_a_ × climate interactions and therefore in understanding effects of elevated CO_2_ concentrations. This contribution is particularly large when tree‐ring studies focus on poorly represented biomes, include representative trees, include recent growth rings, are interpreted cautiously, and are shared in public databases. Our approach can be extended with additional wood‐based measurements that represent tree responses to *c*_a_ × climate interactions: ^13^C isotope analyses (to infer stomatal response) and wood anatomical measurements (to infer tree hydraulic responses).

Our results suggest that effect of CO_2_ rise in DGVMs may be overestimated as these generally assume positive *c*_a_ × T interactions and negative *c*_a_ × P interactions, both of which likely enhance growth. For warmer sites and during warm seasons at cooler sites, we also found the opposite interactions, suggesting that growth reductions may occur under elevated *c*_a_ during warm and dry years. Our results provide opportunities to benchmark tree‐growth rates predicted by vegetation models (Clark et al., 2017). In addition, our framework of hypothesized *c*_a_ × climate interactions can be used to verify the geographic extent and climatic conditions for which NPP (or NPP_stem_) predictions of DGVMs agree with the four hypothesized interactions. We conclude that a better representation of the complex *c*_a_ × climate interactions in DGVMs is likely needed to reduce uncertainty in predicting tropical forest responses to future *c*_a_ rise and climatic change.

## Supporting information

Supplementary Material

## Data Availability

Data sharing not applicable—no new data generated. Tree‐ring data for the two Australian sites are available in the ITRDB (https://www.ncdc.noaa.gov/data‐access/paleoclimatology‐data/datasets/tree‐ring).
