# Supplementary material for: Recent CO2 rise has modified the sensitivity of tropical tree growth to rainfall and temperature
Source: Glob Chang Biol. 2020 May 22;26(7):4028–41. doi: 10.1111/gcb.15092 (PMC7317543; doi:10.1111/gcb.15092)
Supplement: Supplementary file 1 — Supplementary Material [file GCB-26-4028-s001.pdf]

Supporting Information

Recent CO_2 rise has modified the sensitivity of tropical tree growth to rainfall and temperature

Pieter A. Zuidema, Ingo Heinrich, Mizanur Rahman, Mart Vlam, Sophie A. Zwartsenberg & Peter van der Sleen

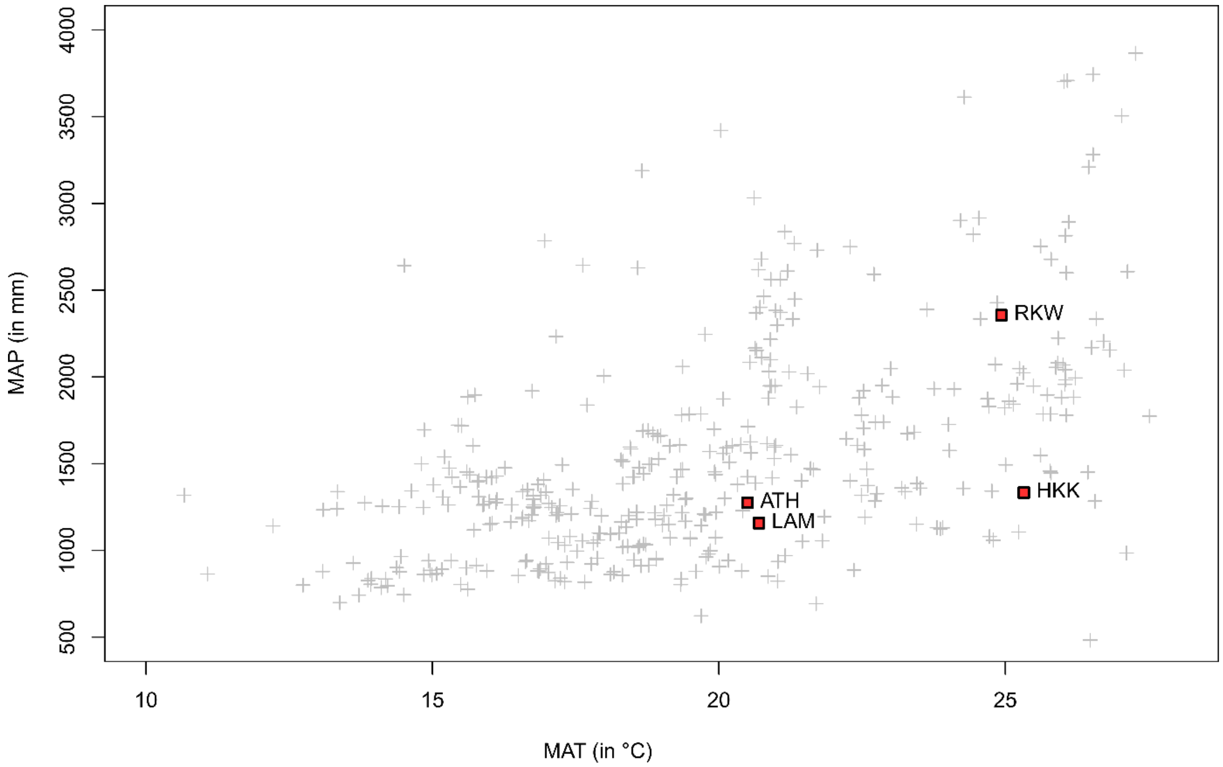

Figure S1. Sample sites (red squares) shown in the Mean Annual Precipitation (MAP) and Mean Annual Temperature (MAT) space. The grey crosses represent herbarium accession of *Toona ciliata* available on GBIF (GBIF.org, 2019). MAT and MAP was calculated using WorldClim 2.0 data (www.worldclim.org) (Fick & Hijmans, 2017).

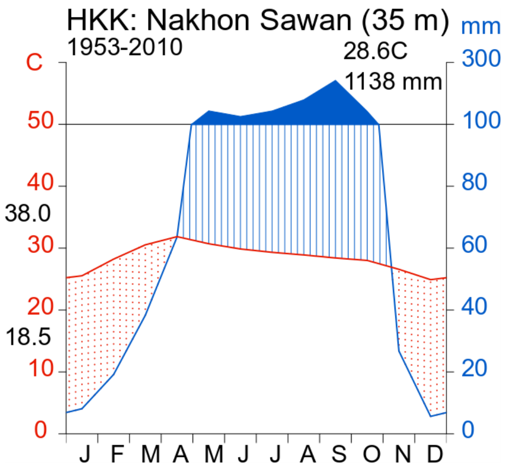

Figure S2. Climate diagram for Nakhon Sawan station. Climate data from this station were used in climate-growth analyses for the HKK site. MAT of this station is approximately 5˚C higher than at the HKK site, due to a 500 m lower elevation.

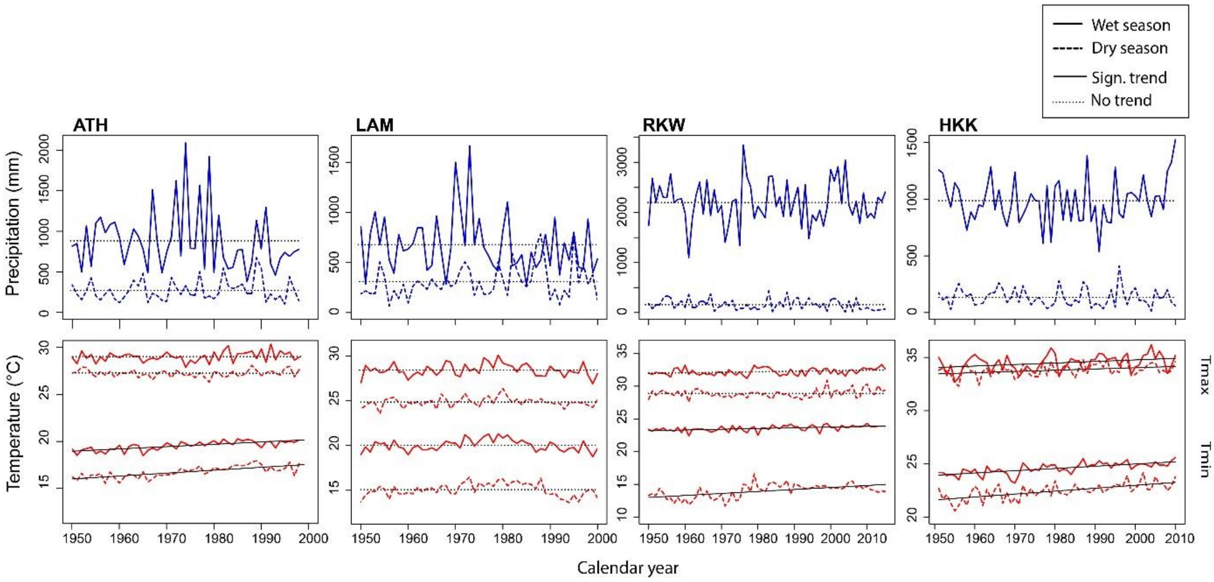

Figure S3. Climate data used in statistical analyses of climate-growth relations and c_a x climate interactions. Data are shown for Wet and Dry seasons (as defined in Table 1) for the period in which both climate and tree-ring data were available. Linear trends were evaluated using simple linear regressions: continuous lines represent significant trends ($p<0.05$); dashed lines non-significant relations. Significant temperature change during the study period was found for ATH: T_{min} +1.2˚C and +1.5˚C (wet and dry season); RKW: T_{min} +0.7 and +2.1˚C (wet and dry season) and HKK: T_{max} +0.7 and +0.9˚C (wet and dry season); T_{min} +1.2 and +1.7˚C (wet and dry season).

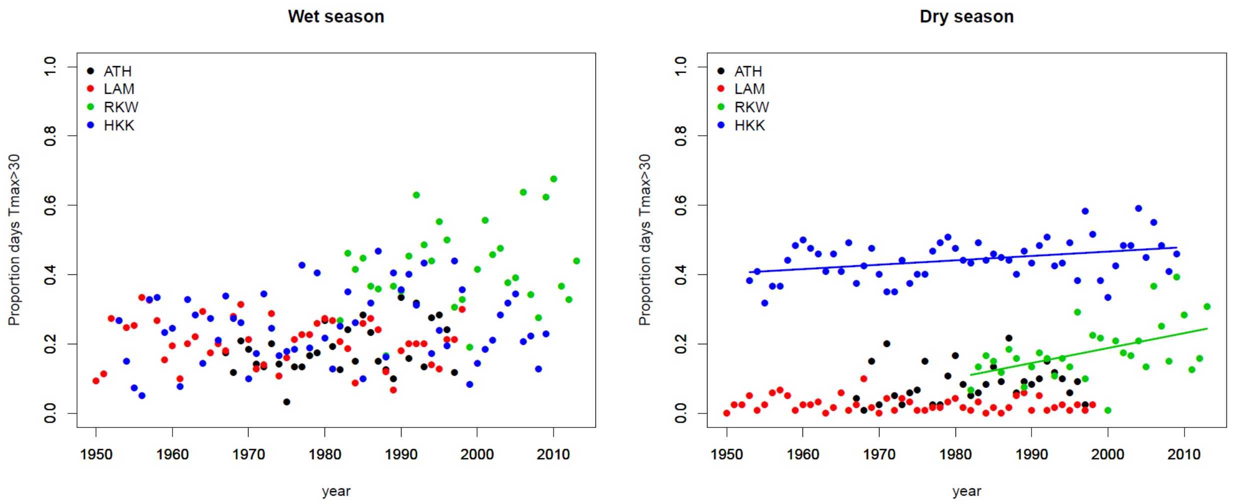

Figure S4. Analyses of the proportion of days with likely above-optimum temperature for photosynthesis. Shown is the proportion of days during the wet or dry season during which T_{max} exceeds 30˚C, leading to leaf temperatures of >32˚C, which is above optimum temperature for photosynthesis (Mau, Reed, Wood, & Cavaleri, 2018). Linear trends were evaluated using simple linear regressions, and if significant, represented by a continuous line ($p<0.05$). A significant increase in number of days >30˚C was found for HKK and RKW in the dry season. For HKK: a threshold temperature of 35˚C was used as climate data come from the low-elevation Nakhon Sawan station, which has a 5˚C higher MAT than the HKK site. For RKW, data from a nearby station (Osmany Int) were used, as daily T_{max} values were unavailable for the Sreemangal station used in our mixed effect models.

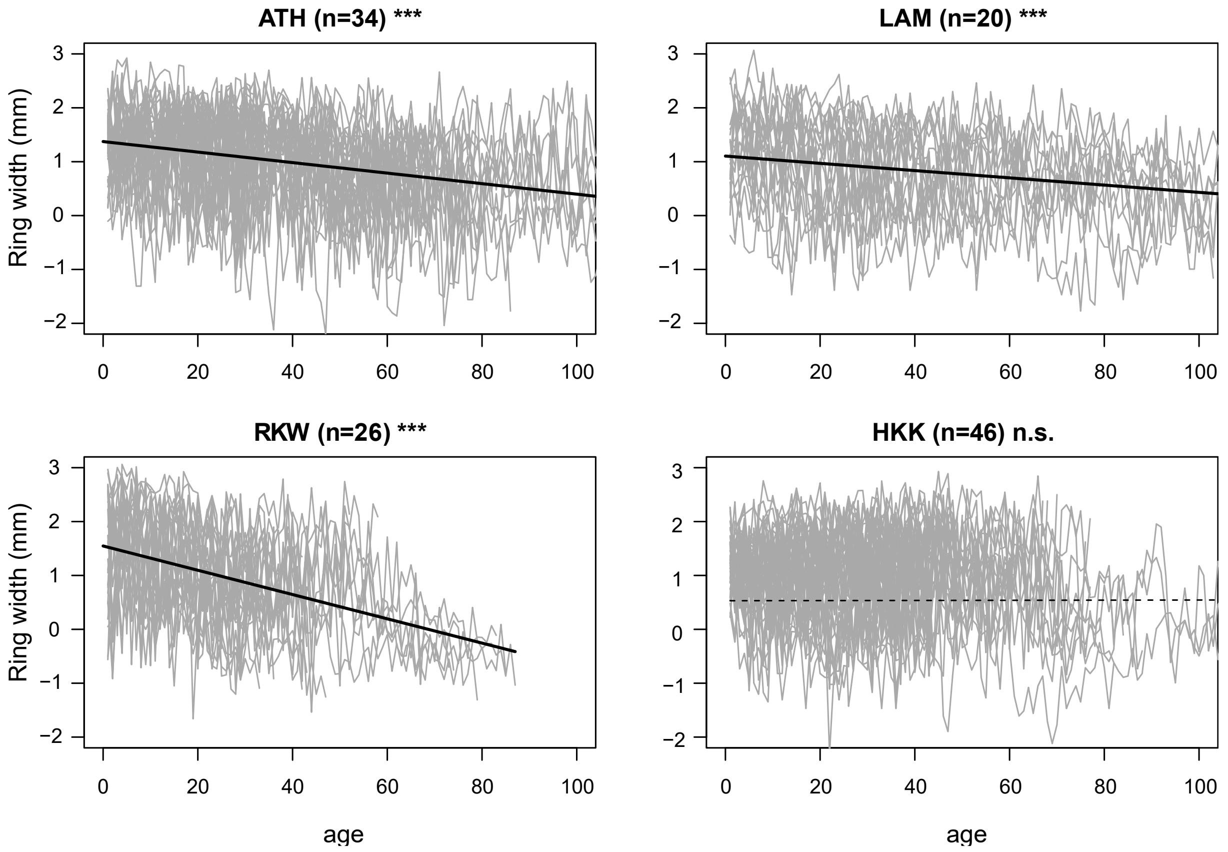

Figure S5. Raw ring-width series (log-transformed) of *Toona ciliata* trees (grey lines) vs estimated age at ring formation at the four study sites. Site abbreviations are explained in Table 1; sites are ordered with increasing mean annual temperature. Overall trends indicated with back line (solid for significant trends, dotted for non-significant, with stars after site name indicating the significance level: *<0.05, **<0.01, ***<0.001, n.s. = not significant).

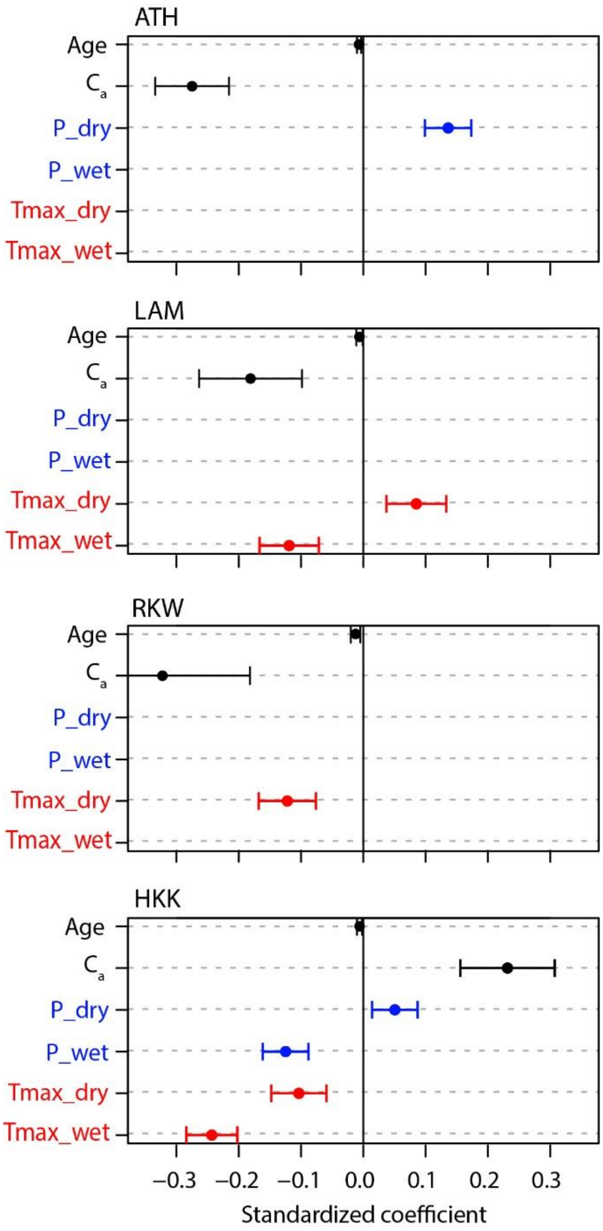

Figure S6. Standardized coefficients obtained from mixed effect models (set A) in which only effects of climate variables on *Toona ciliata* ring width were evaluated, for four sites. Climatic variables included in the analyses are seasonal precipitation (P) and maximum temperature (T_{max}). Summary of model parameters is included in Table 2. Sites ATH and LAM are cooler; RKW and HKK are warmer.

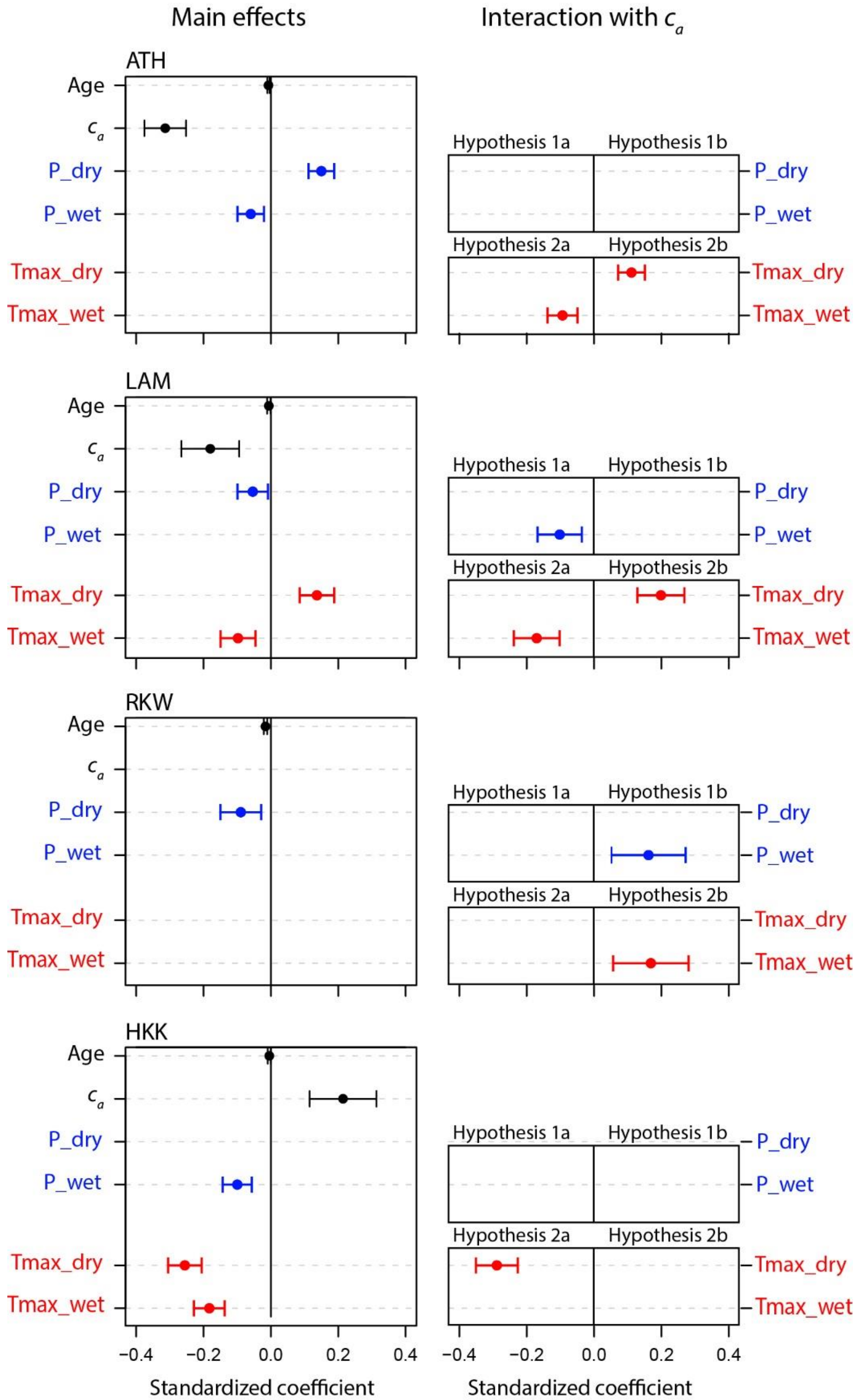

Figure S7. Results from mixed effect models (set C) analysing drivers of *Toona ciliata* ring width, for four study sites, over the common period 1953-1998. Shown are standardized coefficients (mean and 95% confidence interval) of main climate effects and c_a x climate interactions. Tested seasonal climate variables are seasonal precipitation (P, blue) and maximum temperature (T_{max}, red).

Table S1. Characteristics of the published tree-ring width chronologies for *Toona ciliata* used in this study. Shown are number of trees (#Trees) and radii (#Radii), period covered, mean interseries correlation (Rbar), mean sensitivity (MSI) and expressed population signal (EPS). MSI is a measurement of the year-to-year variability in tree-ring width. The EPS evaluates to what degree a tree-ring chronology based on a sample of trees estimates the hypothetical (noise-free) population chronology. The shown EPS values are for the full periods covered by the chronologies.

Site code	#Trees	#Radii	Period	Rbar	MSI	EPS	Climate station	Reference
ATH	37	53	1860-2000	0.52	0.60	>0.85	Kairi	(Heinrich, Weidner, Helle, Vos, & Banks, 2008)
LAM	20	52	1854-2000	0.54	0.59	>0.85	Brisbane	(Heinrich et al., 2009)
RKW	26	40	1930-2015	0.27	0.49	>0.85	Sreemangal	(Rahman, Islam, & Bräuning, 2018)
HKK	46	168	1950-2011	0.26	0.50	>0.85	Nakhon Sawan	(Vlam, Baker, Bunyavejchewin, & Zuidema, 2014)

Table S2. Correlations between precipitation (P) and T_{max} or T_{min} during wet and dry season. Pearson correlation coefficients (*r*) and significance. Significance levels: NS, *: $p<0.05$; **: $p<0.01$; ***: $p<0.001$.

Site code	Wet season: P vs T_{max}	Wet season: P vs T_{min}	Dry season: P vs T_{max}	Dry season: P vs T_{min}
ATH	-0.50***	NS	NS	0.55***
LAM	-0.32*	NS	NS	0.44**
RKW	-0.40*	NS	-0.32*	NS
HKK	-0.38***	NS	-0.53***	NS

Table S3. Qualitative review of c_a x climate interaction experiments for tropical (Tro), subtropical (Sub) and temperate (Tem) tree species. Plant sizes are seedling (S) or small trees (Tr) and studies were performed in glasshouses (G) or in a climate controlled field chamber (CC). Codes 1a-2b refer to significant interactions supporting hypotheses in Figure 1; NS = non-significant interaction. Results from Scopus search string: TITLE-ABS-KEY (climate AND forest AND CO2 AND growth)) AND (tropic), and papers citing Lloyd and Farquhar (2008), until July 2019.

								**c_a x water**			**c_a x T**			
**Species**	**Climate zone**	**Plant size**	**Study type**	**c_a treatment**	**Water treatments**	**Temperature treatments (˚C)**	**Response variable(s)**	**1a**	**1b**	**NS**	**2a**	**2b**	**NS**	**Reference**
Swietenia macrophylla	Tro	S	G	40/70 Pa	FC/30%	max 30	Whole plant dry mass			x				Cernusak et al. (2011)
Ormosia macrocalyx	Tro	S	G	40/70 Pa	FC/30%	max 30	Whole plant dry mass			x				Cernusak et al. (2011)
Carapa surinamensis	Tro	S	G	400/700 ppm	FC/50%	-	Light saturated photosynthesis	x						M.F. de Oliveira and R.A. Marenco (2019)
Eucalyptus populnea	Tro	S	G	380/ 700 umol/mol	FC/50%	25 day and 16 night	Whole plant dry mass			x				Kelly, Duursma, Atwell, Tissue, and Medlyn (2016)
Eucalyptus pilularis	Tro	S	G	380/ 700 umol/mol	FC/50%	25 day and 16 night	Whole plant dry mass			x				Kelly et al. (2016)
Alchornea glandulosa	Tro	S	G	Ambient/ 800 ppm	-	Ambient/Ambient +1.5	Net photosynthesis					x		Fauset et al. (2019)
Carapa surinamensis	Tro	S	G	400/700 ppm	FC/50%	-	Light saturated photosynthesis	x						(M. F. de Oliveira & R. A. Marenco, 2019)
Eucalyptus saligna	Sub	S	G	290/400/ 650 uL/L	-	Simulated 30 yr average/+4	Whole plant dry mass					x		Ghannoum et al. (2010)

								c_a x water			c_a x T			
**Species**	**Climate zone**	**Plant size**	**Study type**	**c_a treatment**	**Water treatments**	**Temperature treatments (˚C)**	**Response variable(s)**	**1a**	**1b**	**N S**	**2a**	**2b**	**N S**	**Reference**
Eucalyptus sideroxylon	Sub	S	G	290/400/ 650 uL/L	-	Simulated 30 yr average/+4	Whole plant dry mass					x		Ghannoum et al. (2010)
Eucalyptus saligna	Sub	S	G	290/400/ 650 uL/L	Daily watering/ max 200g water loss daily, when wilting re-watering till saturation	Ambient/ Ambient +4	Light saturated photosynthesis and stem dry mass	x				x		Lewis et al. (2013)
Eucalyptus sideroxylon	Sub	S	G	290/400/ 650 uL/L	Daily watering/ max 200g water loss daily, when wilting re-watering till saturation	Ambient/ Ambient +4	Light saturated photosynthesis and stem dry mass			x		x		Lewis et al. (2013)
Eucalyptus saligna	Sub	S	G	290/400/ 650 uL/L	-	Ambient/ Ambient +4	Light saturated photosynthesis					x		Logan et al. (2010)
Eucalyptus sideroxylon	Sub	S	G	290/400/ 650 uL/L	-	Ambient/ Ambient +4	Light saturated photosynthesis						x	Logan et al. (2010)
Callitris rhomboidea	Tem	S	G	400/640 umol/mol	FC/Progressive drought till mortality	Ambient/ Ambient +4	Light saturated photosynthesis and dry mass		x				x	Duan et al. (2015)
Pinus radiata	Tem	S	G	400/640 umol/mol	FC/Progressive drought till mortality	Ambient/ Ambient +4	Light saturated photosynthesis and dry mass			x			x	Duan et al. (2015)
Callitris rhomboidea	Tem	S	G	400/640 umol/mol	FC/Progressive drought till mortality	Ambient/ Ambient +4	Whole plant dry mass			x		x		Duan, Huang, Zhou, and Tissue (2018)

								c_a x water			c_a x T			
**Species**	**Climate zone**	**Plant size**	**Study type**	c_a **treatment**	**Water treatments**	**Temperature treatments (˚C)**	**Response variable(s)**	**1a**	**1b**	**NS**	**2a**	**2b**	**NS**	**Reference**
Pinus radiata	Tem	S	G	400/640 umol/mol	FC/Progressive drought till mortality	Ambient/ Ambient +4	Whole plant dry mass			x			x	Duan et al. (2018)
Eucalyptus globulus	Tem	Tr	CC	Ambient/+ 240ppm	-	Ambient/ Ambient +3	Net photosynthesis and whole tree dry mass						x	Quentin, Barton, Crous, and Ellsworth (2015)
Betula papyrifera	Tem	S	G	360/720 umol/mol		7, 17, 27 (soil temperatures)	Net photosynthesis					x		Ambebe, Dang, and Li (2009)

References

Ambebe, T. F., Dang, Q. L., & Li, J. (2009). Low soil temperature inhibits the effect of high nutrient supply on photosynthetic response to elevated carbon dioxide concentration in white birch seedlings. *Tree Physiology, 30*(2), 234-243. doi:10.1093/treephys/tpp109

Cernusak, L. A., Winter, K., Martínez, C., Correa, E., Aranda, J., Garcia, M., . . . Turner, B. L. (2011). Responses of legume versus nonlegume tropical tree seedlings to elevated CO2 concentration. *Plant Physiology, 157*(1), 372-385. doi:10.1104/pp.111.182436

de Oliveira, M., & Marenco, R. (2019). Gas exchange, biomass allocation and water-use efficiency in response to elevated CO2 and drought in andiroba (Carapa surinamensis, Meliaceae). [Gas exchange, biomass allocation and water-use efficiency in response to elevated CO2 and drought in andiroba (Carapa surinamensis, Meliaceae)]. *iForest - Biogeosciences and Forestry, 12*(1), 61-68. doi:10.3832/ifor2813-011

de Oliveira, M. F., & Marenco, R. A. (2019). Photosynthesis and biomass accumulation in carapa surinamensis (Meliaceae) in response to water stress at ambient and elevated CO2. *Photosynthetica, 57*(1), 137-146. doi:10.32615/ps.2019.023

Duan, H., Huang, G., Zhou, S., & Tissue, D. T. (2018). Dry mass production, allocation patterns and water use efficiency of two conifers with different water use strategies under elevated [CO2], warming and drought conditions. *European Journal of Forest Research, 137*(5), 605-618. doi:10.1007/s10342-018-1128-x

Duan, H., O'Grady, A. P., Duursma, R. A., Choat, B., Huang, G., Smith, R. A., . . . Tissue, D. T. (2015). Drought responses of two gymnosperm species with contrasting stomatal regulation strategies under elevated [CO2] and temperature. *Tree Physiology, 35*(7), 756-770. doi:10.1093/treephys/tpv047

Fauset, S., Oliveira, L., Buckeridge, M. S., Foyer, C. H., Galbraith, D., Tiwari, R., & Gloor, M. (2019). Contrasting responses of stomatal conductance and photosynthetic capacity to warming and elevated CO2 in the tropical tree species Alchornea glandulosa under heatwave conditions. *Environmental and Experimental Botany, 158*, 28-39. doi:https://doi.org/10.1016/j.envexpbot.2018.10.030

Fick, S. E., & Hijmans, R. J. (2017). WorldClim 2: new 1-km spatial resolution climate surfaces for global land areas. *International Journal of Climatology, 37*(12), 4302-4315. doi:10.1002/joc.5086

Ghannoum, O., Phillips, N. G., Conroy, J. P., Smith, R. A., Attard, R. D., Woodfield, R., . . . Tissue, D. T. (2010). Exposure to preindustrial, current and future atmospheric CO2 and temperature differentially affects growth and photosynthesis in Eucalyptus. *Global Change Biology, 16*(1), 303-319. doi:10.1111/j.1365-2486.2009.02003.x

Ambebe, T. F., Dang, Q. L., & Li, J. (2009). Low soil temperature inhibits the effect of high nutrient supply on photosynthetic response to elevated carbon dioxide concentration in white birch seedlings. *Tree Physiology, 30*(2), 234-243. doi:10.1093/treephys/tpp109

Cernusak, L. A., Winter, K., Martínez, C., Correa, E., Aranda, J., Garcia, M., . . . Turner, B. L. (2011). Responses of legume versus nonlegume tropical tree seedlings to elevated CO2 concentration. *Plant Physiology, 157*(1), 372-385. doi:10.1104/pp.111.182436

de Oliveira, M. F., & Marenco, R. A. (2019). Gas exchange, biomass allocation and water-use efficiency in response to elevated CO2 and drought in andiroba (Carapa surinamensis, Meliaceae). [Gas exchange, biomass allocation and water-use efficiency in response to elevated CO2 and drought in andiroba (Carapa surinamensis, Meliaceae)]. *iForest - Biogeosciences and Forestry, 12*(1), 61-68. doi:10.3832/ifor2813-011

de Oliveira, M. F., & Marenco, R. A. (2019). Photosynthesis and biomass accumulation in carapa surinamensis (Meliaceae) in response to water stress at ambient and elevated CO2. *Photosynthetica, 57*(1), 137-146. doi:10.32615/ps.2019.023

Duan, H., Huang, G., Zhou, S., & Tissue, D. T. (2018). Dry mass production, allocation patterns and water use efficiency of two conifers with different water use strategies under elevated [CO2], warming and drought conditions. *European Journal of Forest Research, 137*(5), 605-618. doi:10.1007/s10342-018-1128-x

Duan, H., O'Grady, A. P., Duursma, R. A., Choat, B., Huang, G., Smith, R. A., . . . Tissue, D. T. (2015). Drought responses of two gymnosperm species with contrasting stomatal regulation strategies under elevated [CO2] and temperature. *Tree Physiology, 35*(7), 756-770. doi:10.1093/treephys/tpv047

Fauset, S., Oliveira, L., Buckeridge, M. S., Foyer, C. H., Galbraith, D., Tiwari, R., & Gloor, M. (2019). Contrasting responses of stomatal conductance and photosynthetic capacity to warming and elevated CO2 in the tropical tree species Alchornea glandulosa under heatwave conditions. *Environmental and Experimental Botany, 158*, 28-39. doi:https://doi.org/10.1016/j.envexpbot.2018.10.030

Fick, S. E., & Hijmans, R. J. (2017). WorldClim 2: new 1-km spatial resolution climate surfaces for global land areas. *International Journal of Climatology, 37*(12), 4302-4315. doi:10.1002/joc.5086

GBIF.org. (2019). *GBIF Occurrence Download*. Retrieved from: https://doi.org/10.15468/dl.ijp7dz

Ghannoum, O., Phillips, N. G., Conroy, J. P., Smith, R. A., Attard, R. D., Woodfield, R., . . . Tissue, D. T. (2010). Exposure to preindustrial, current and future atmospheric CO2 and temperature differentially affects growth and photosynthesis in Eucalyptus. *Global Change Biology, 16*(1), 303-319. doi:10.1111/j.1365-2486.2009.02003.x

Heinrich, I., Weidner, K., Helle, G., Vos, H., & Banks, J. C. G. (2008). Hydroclimatic variation in Far North Queensland since 1860 inferred from tree rings. *Palaeogeography, Palaeoclimatology, Palaeoecology, 270*(1-2), 116-127. doi:10.1016/j.palaeo.2008.09.002

Heinrich, I., Weidner, K., Helle, G., Vos, H., Lindesay, J., & Banks, J. C. G. (2009). Interdecadal modulation of the relationship between ENSO, IPO and precipitation: insights from tree rings in Australia. *Climate Dynamics, 33*(1), 63-73. doi:10.1007/s00382-009-0544-5

Kelly, J. W. G., Duursma, R. A., Atwell, B. J., Tissue, D. T., & Medlyn, B. E. (2016). Drought × CO_2 interactions in trees: a test of the low-intercellular CO_2 concentration (Ci) mechanism. *New Phytologist, 209*(4), 1600-1612. doi:doi:10.1111/nph.13715

Lewis, J. D., Smith, R. A., Ghannoum, O., Logan, B. A., Phillips, N. G., & Tissue, D. T. (2013). Industrial-age changes in atmospheric [CO_2] and temperature differentially alter responses of faster- and slower-growing Eucalyptus seedlings to short-term drought. *Tree Physiology, 33*(5), 475-488. doi:10.1093/treephys/tpt032

Lloyd, J., & Farquhar, G. D. (2008). Effects of rising temperatures and [CO_2] on the physiology of tropical forest trees. *Philosophical Transactions of the Royal Society B: Biological Sciences, 363*(1498), 1811-1817. doi:10.1098/rstb.2007.0032

Logan, B. A., Hricko, C. R., Lewis, J. D., Ghannoum, O., Phillips, N. G., Smith, R., . . . Tissue, D. T. (2010). Examination of pre-industrial and future [CO_2] reveals the temperature-dependent CO_2 sensitivity of light energy partitioning at PSII in eucalypts. *Functional Plant Biology, 37*(11), 1041-1049. doi:10.1071/FP10113

Mau, A., Reed, S., Wood, T., & Cavaleri, M. (2018). Temperate and tropical forest canopies are already functioning beyond their thermal thresholds for photosynthesis. *Forests, 9*(1), 47.

Quentin, A. G., Barton, C. V. M., Crous, K. Y., & Ellsworth, D. S. (2015). Photosynthetic enhancement by elevated CO_2 depends on seasonal temperatures for warmed and non-warmed Eucalyptus globulus trees. *Tree Physiology, 35*(11), 1249-1263. doi:10.1093/treephys/tpv110

Rahman, M., Islam, M., & Bräuning, A. (2018). Tree radial growth is projected to decline in South Asian moist forest trees under climate change. *Global and Planetary Change, 170*, 106-119. doi:https://doi.org/10.1016/j.gloplacha.2018.08.008

Vlam, M., Baker, P. J., Bunyavejchewin, S., & Zuidema, P. A. (2014). Temperature and rainfall strongly drive temporal growth variation in Asian tropical forest trees. *Oecologia, 174*(4), 1449-1461. doi:10.1007/s00442-013-2846-x
